# Gavi's Zero-Dose Learning Hubs: enhanced approach for evidence generation and use from local to global

**DOI:** 10.1093/heapol/czag028

**Published:** 2026-03-10

**Authors:** Heidi W Reynolds, Gustavo C Corrêa, Nancy Vollmer, Erin Broekhuysen, Esther Saville, Dan Hogan, Hope L Johnson

**Affiliations:** Measurement, Evaluation, and Learning Department, Gavi, The Vaccine Alliance, Chemin du Pommier 40, Le Grand Saconnex 1218, Switzerland; Measurement, Evaluation, and Learning Department, Gavi, The Vaccine Alliance, Chemin du Pommier 40, Le Grand Saconnex 1218, Switzerland; John Snow Research and Training, Inc, 2733 Crystal Dr 4th Floor, Arlington, VA 22202, United States; John Snow Research and Training, Inc, 2733 Crystal Dr 4th Floor, Arlington, VA 22202, United States; Measurement, Evaluation, and Learning Department, Gavi, The Vaccine Alliance, Chemin du Pommier 40, Le Grand Saconnex 1218, Switzerland; Measurement, Evaluation, and Learning Department, Gavi, The Vaccine Alliance, Chemin du Pommier 40, Le Grand Saconnex 1218, Switzerland; Measurement, Evaluation, and Learning Department, Gavi, The Vaccine Alliance, Chemin du Pommier 40, Le Grand Saconnex 1218, Switzerland

**Keywords:** zero-dose children, implementation research, immunization, vaccination, learning, evaluation, health systems, Bangladesh, Mali, Nigeria, Uganda

## Abstract

Reaching and fully immunizing zero-dose (ZD) children and missed communities is at the core of the Gavi, The Vaccine Alliance 5.0/5.1 and Immunization Agenda 2030 strategies. This is critical to ensure equitable immunization coverage and access to other primary health care services and to prevent outbreaks. The diversity of settings where these children live and the complexity of vaccination barriers require a complementary set of activities embedded in national systems. Learning approaches are needed to use evidence to improve equity and reach. Gavi has helped fill this gap with the Zero-Dose Learning Hub (ZDLH) initiative, which is composed of consortia partners in four countries—Mali, Nigeria, Uganda, and Bangladesh—and a global-level consortium. This paper describes the ZDLH design, theory of change, methods, and measures of success. Then, future papers will present ZDLH results, successes and challenges, and recommendations. The ZDLH initiative is prospective and runs through 2025. It features primary evidence generation through rapid assessments, improved monitoring, implementation research in targeted subnational areas where ZD children are located, and country-specific learning agendas and knowledge translation activities to facilitate evidence use. The global-level consortium offers technical assistance to country learning hubs and facilitates synthesis, dissemination, and improves evidence use across low- and middle-income countries. A common measurement, evaluation, and learning plan documents whether evidence is generated and used and how the overall model works to inform future adaptation. Target audiences for evidence are the Gavi Board; Gavi strategy, programme, and country teams; countries’ ministry of health and immunization programmes at national and subnational level; and other donors and implementing partners working to improve immunization equity. The ZDLH initiative is a coherent approach to evidence generation and learning, and the implementation experience informs how to better design and support learning systems embedded within National Health Systems.

Key messages

- To improve immunization coverage and primary health care access for zero-dose (ZD) children and missed communities—who are consistently overlooked due to persistent barriers and a lack of straightforward solutions—we adopted a learning hub model. This model is designed for practical implementation and rapid delivery of timely, actionable insights.
- The Zero-Dose Learning Hub (ZDLH) initiative is composed of consortia in four countries and a global-level learning partner consortium.
- The ZDLH initiative features primary evidence generation and use activities through rapid assessments, improved monitoring of key outcomes, implementation research, and country-specific learning agendas, and knowledge translation.
- The learning hubs are a coherent approach to evidence generation and learning, and the implementation experience serves to inform how to better design and support learning systems embedded within National Health Systems.

## Introduction

While there have been important gains in vaccine coverage and the introduction of new vaccines over the past 50 years since the launch of the Expanded Programme on Immunization (EPI), progress has stalled, and those most in need—particularly zero-dose (ZD) children—continue to be systematically missed (Hogan and Gupta 2023). Prior to the Covid-19 pandemic, between 2010 and 2019, increases in vaccine coverage measured by the third dose of diphtheria-tetanus-pertussis (DTP)-containing vaccine (DTP3) slowed, increasing only three percentage points globally during that time, whereas the coverage of the first dose of DTP-containing vaccine (DTP1) increased by only one percentage point. This means increases in DTP3 coverage were largely due to children completing their second and third doses in the DPT series (Hogan and Gupta 2023). Therefore, reaching children with their first dose of DTP is critical to improving overall immunization coverage and reducing under-five mortality (Hogan and Gupta 2023), and it should confer several other benefits including increasing access to full immunization and primary health care (Santos et al. 2021).

A zero-dose child is one who has missed out on receiving any routine immunizations; however, this can typically only be measured through household surveys as health management information systems often do not track individual-level vaccination status. To enable more routine tracking of progress on reaching ZD children, the IA2030 Framework for Action identified DTP1 as a correlated and actionable metric (IA 2030 2020). Gavi operationally defines zero-dose children as those children who have not received their first dose of DTP-containing vaccine, including the pentavalent vaccine (IA 2030 2023), although the practical meaning is to find and reach children and communities that have been systematically missed by immunization services. The concept represents acute inequities that must be directly addressed within affected communities (ERG 2020).

There is no one-size-fits-all solution to identify and reach zero-dose children. Un- or under-immunized children are more likely to be concentrated in urban slums, remote rural, or conflict-affected geographies (ERG 2020, Wigley et al. 2022) and face multiple deprivations (Santos et al. 2021, Wendt et al. 2022), including those posed by ethnic discrimination or gender-related factors such as low maternal education, limited autonomy to make household decisions, or barriers specific to female caregivers (e.g. time and workload constraints, mobility and safety concerns) (Chopra et al. 2020 ). There is also variation in the determinants associated with those deprivations (Cata-Preta et al. 2022, Wendt et al. 2022). The Board of Gavi, the Vaccine Alliance acknowledged the lack of evidence and solutions to effectively reach children and communities systematically missed for generations and, in December 2020, recommended establishing a set of learning priorities to align monitoring, evaluation, and other learning activities (Gavi 2020, 2024a). Gavi adopted an “execution-as-learning” approach, which is the practice of regularly implementing interventions, generating evidence and learning, and adapting rapidly. This approach, central to the 2021–2025 strategy (Gavi 5.0) and the Immunization Agenda 2030 (IA2030), led to the creation of the Zero-Dose Learning Hubs (ZDLH) (Immunisation Agenda [IA] 2030 2020, Gavi 2020, 2021b, 2022).

The ZDLH initiative moves beyond traditional monitoring and evaluation to approaches that examine implementation within National Health Systems and bridge the know-do-gap to inform policy (Bennett and Jessani 2021). The ZDLH is established through local partnerships in four countries to supplement routine immunization monitoring and use multiple methods to understand factors influencing implementation and performance of approaches to reach zero-dose and under-immunized children and missed communities.

The purpose of this paper is to describe the ZDLH design, methods, and measures of success to inform efforts that support learning and evidence generation in the context of national systems. This paper will serve as a reference when future data, evidence, and lessons learned are available.

## Methods

### Design

The learning priorities and ZDLH approach are positioned within Gavi’s Learning System Strategy and were based on cross-Alliance consultations and aligned with the Identify-Reach-Monitor-Measure-Advocate (IRMMA) framework (Gavi 2021a, 2022). Questions are intended to be answered across a diversity of settings (remote rural, urban poor, and conflict affected) with a cross-cutting focus on gender equity (ERG 2020). A cross-secretariat working group was established to guide the prioritization and implementation of targeted activities to answer key questions and interpret, synthesize, and leverage findings and recommendations.

The Learning Hubs are multi-year, multi-country, prospective projects that use multidisciplinary mixed methods for evidence generation. The design elements of the country learning hubs (CLH) are summarized in Table 1 and were informed by previous evaluation experiences that offered complementary insights into embedding learning within programme cycles. From 2013 to 2017, Gavi implemented full country evaluations (FCEs) to evaluate how Gavi investments resulted in immunization programme outcomes in Bangladesh, Mozambique, Uganda, and Zambia (Soi et al. 2020). From 2017 to 2019, The Global Fund implemented prospective country evaluations (PCEs) in eight countries: Cambodia, Democratic Republic of Congo, Guatemala, Mozambique, Myanmar, Senegal, Sudan, and Uganda (Carnahan et al. 2020). While the Gavi experience was directly relevant, Global Fund’s PCEs provided valuable lessons because both organizations operate across many low- and middle-income settings, use catalytic financing, and face similar challenges in translating global policies into country-level implementation. Recommendations from these experiences included engaging stakeholders to inform design and interpretation, aligning on the level of engagement embeddedness in programme development, adapting evaluations according to organizational or contextual changes, tailoring recommendations to contexts and communicating underlying strength of evidence, and using mixed methods. Both models also featured country-level partners leading data collection, analysis, and reporting with global-level providers overseeing implementation and synthesizing cross-country findings.

**Table 1:** ZDLH design elements to facilitate the translation of evidence into results for immunization equity.

Design element	Approach
Objectives	Generate learnings based on knowledge of the barriers to reach ZD children and apply these to programme planning and tailoring equitable approaches. Strengthen the evidence base of effective approaches to identify and reach ZD children. Improve metrics, measures, and methods to access and use data on a regular basis to improve outreach to ZD children and missed communities.
Timeframe	Prospective (2022–2025)
Knowledge paradigm and disciplines	Learning health systems, embedded implementation research, realist evaluation
Scope	Policy and programme shifts that enable the implementation of IRMMA-aligned approaches at the targeted subnational levels and changes in the reach of unimmunized children and DTP1 coverage
Country and subnational area selection criteria	Relatively high numbers and proportions of zero-dose children, with variation in geographic setting, and Gavi-eligible country and targeted subnational funding
Data sources, methods, and analytic approaches	Multiple, mixed
Partnerships	Consortia led by a country-based implementing partner with strong relationships with EPI and ministries of health
Global technical and operation support	Consortium provides tailored capacity support and facilitates sharing and learning across partners and contributes to the global evidence base
Governance	Funding and accountability by the Measurement, Evaluation, and Learning (MEL) department at Gavi, the Vaccine Alliance. An Advisory Committee composed of external experts to advise Gavi on implementation
Generalizability and comparability	TOCs, common metrics, common MEL plan components, similar activities and methods, standardized tools and guidance, and support from the global learning provider consortium
Dissemination and feedback loops	Regular dissemination and feedback to subnational and national level stakeholders, across LH countries via regular meetings and intranet, globally via the global learning provider materials, website (https://zdlh.gavi.org), leveraging the ZD COP and TGLF peer learning events
Users and use cases	Gavi Board monitoring the implementation of the 5.0 strategy; Gavi country teams; country MOH and EPI stakeholders at national and subnational level

The ZDLH model design was also informed by multiple consultations with learning experts and implementers, Gavi country teams and leadership, Alliance partners, and other global and country partners implementing immunization programmes. The objective of the consultations was to collect more in-depth information on the positive enabling experiences, pitfalls to avoid, and gaps and needs, and to ensure that both country and global information needs are met. As potential users of evidence, including these stakeholders was paramount to the design.

The ZLDH design is also driven by the concept of learning health systems for low- and middle-income country settings and decision-maker led implementation research (IR). The concept means that learning is integrated in monitoring and evaluation practices throughout the strategic planning cycle to improve health system functions, support adaptation and innovation, and engender self-reliance (Sheikh and Abimbola 2021). These approaches produce evidence needed to respond, adapt, and improve. A key feature is that country-led stakeholders set the research agenda, prioritize interventions for collaborative research, and ensure use for programmatic improvements, underpinned by capacity development through workshops and mentorship (Ghaffar et al. 2020). While ZDLH is a learning project, the learning hubs feature principles of realist evaluation in that they consider the programme theory and the context in which the intervention is implemented and work to understand how the outcomes were achieved or why not (Better Evaluation 2023).

**Figure 1:**
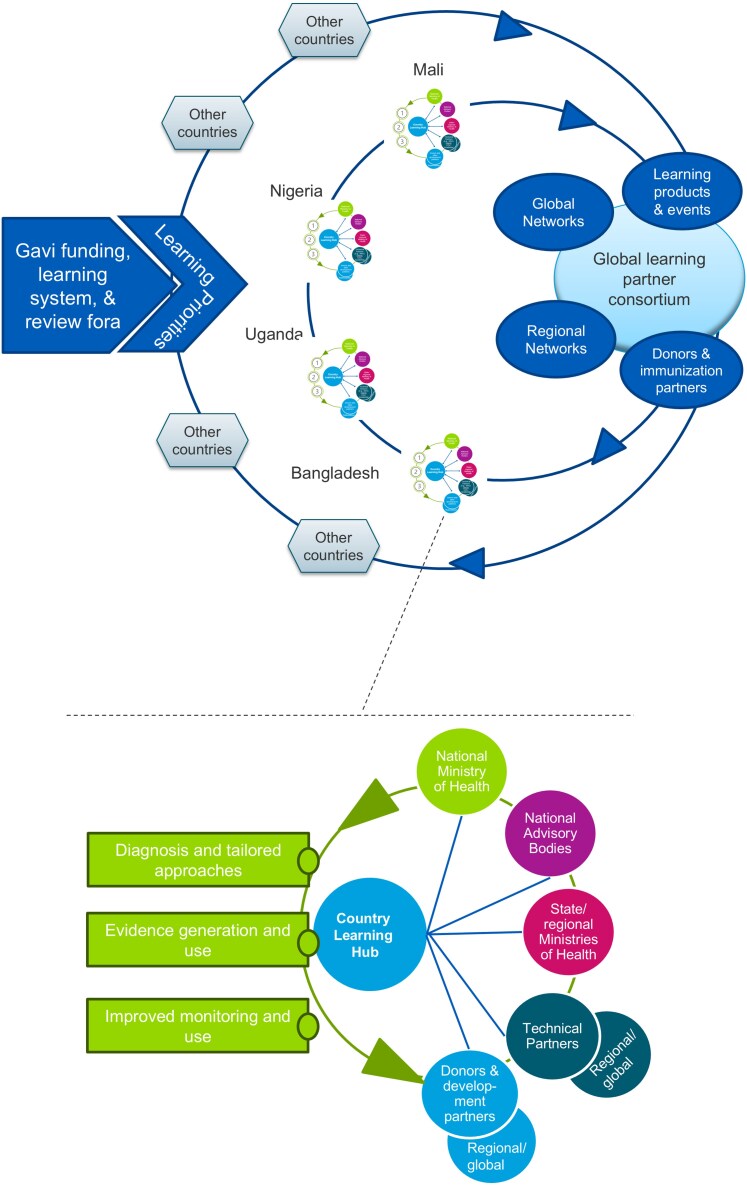
Country and global learning hub structure and function.

Countries (Mali, Nigeria, Bangladesh, and Uganda) were selected for their relatively high numbers and proportions of zero-dose children, diverse intra-country contexts and to ensure geographic variation across the 57 eligible countries for Gavi vaccine support (in 2021; https://www.gavi.org/types-support/sustainability/eligibility). Due to resource constraints, the final selection was limited to four countries. In each country, the Learning Hub is a hub-and-spoke model led by local partners/consortiums (Fig. 1). Although not a physical brick-and-mortar hub, partners regularly engage across government, non-government organizations, and multilaterals to share activities and learning.

At the global level, the consortium provides technical support to CLHs and works to identify, synthesize, and disseminate data and evidence. However, Gavi directly manages CLH contractual arrangements and monitors accountability and performance.

Prior to issuing the request for proposals, Gavi held a sensitization and feedback discussion event with potential bidders to solicit their feedback and questions. All consortia were selected via a competitive proposal bidding process and met technical and financial requirements. There were five separate processes, one for each country and one for the global provider. Proposals were assessed against clear criteria including having in-country presence, adequate technical composition, and networks with county stakeholders, including ministries of health and expanded programme on immunization (EPI).

CLHs are designed as execution-focused platforms that embed learning directly into implementation. Rather than assessing the contribution of Gavi’s financial support, they concentrate on understanding and accelerating policy and programme shifts that enable countries to identify and reach zero-dose children, although these shifts are often supported by Gavi funding. CLHs operate as country-led initiatives with networks of local partners and stakeholders united by a shared purpose: generating evidence and applying it to improve delivery. Embedded within national structures, they use adaptive learning cycles and stakeholder engagement to address priority questions for timely decision-making. Activities within the learning hubs emphasize iterative evidence generation and feedback loops, ensuring that insights are rapidly translated into action to strengthen interventions and close the know-do gap (Box 1).

Box 1. **Stakeholder engagement in Bangladesh**

icddr,b established national and subnational-level fora with participation from officials from the Ministry of Health and Family Welfare (MOHFW), development partners, and other EPI stakeholders who make recommendations for policy based on LH evidence. These groups were also engaged in decisions around intervention design and implementation.

Another characteristic of the CLHs is that much of the focus of learning is in targeted subnational areas with high numbers and proportions of zero-dose children. This feature was considered important due to the assumption that to reach zero-dose children, the interventions must be tailored to the context-specific barriers, and the barriers will vary by context, both geographic (e.g. rural, urban, conflict) and gender with other barriers (e.g. religious, hesitancy).

### Theory of change and objectives

**Figure 2:**
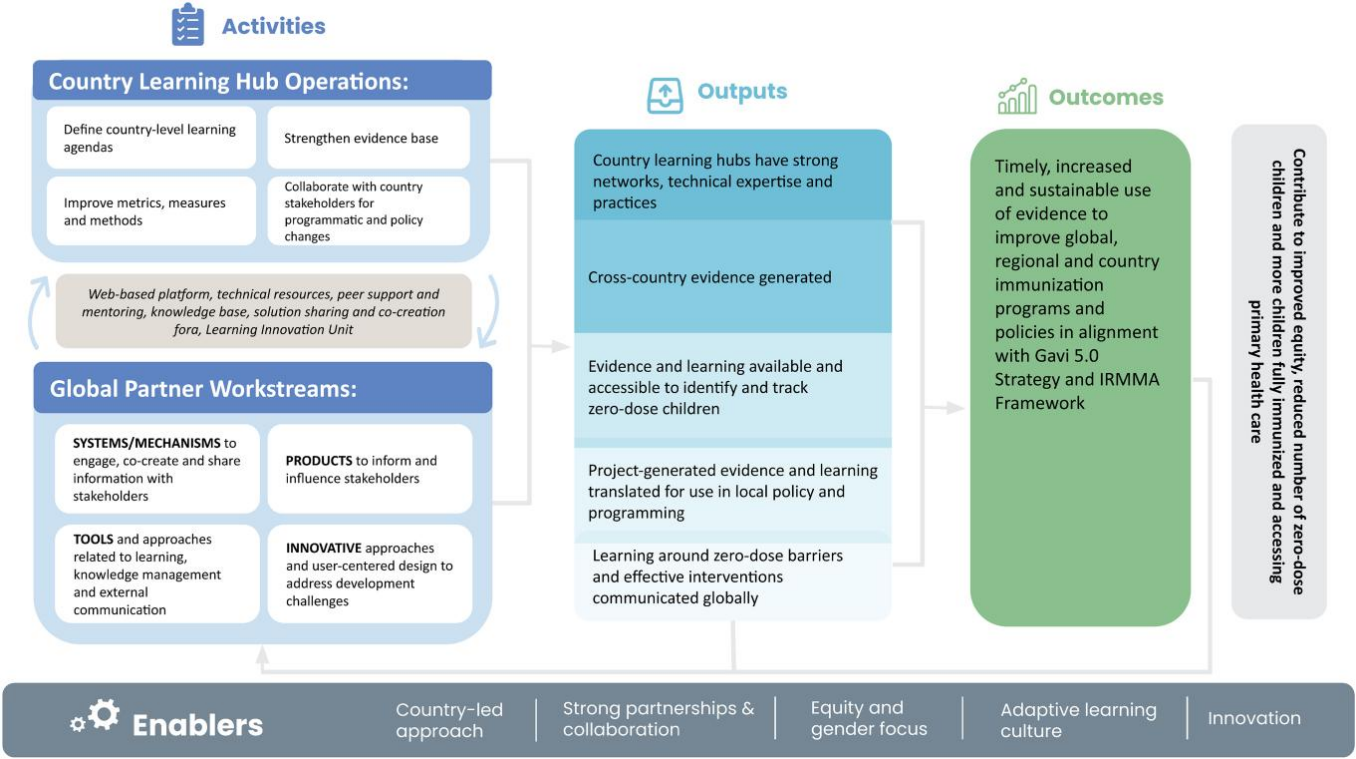
Zero-Dose Learning Hub theory of change.

The CLHs and the global learning partner work together to achieve the overall goal of the ZDLH initiative to improve immunization equity and reduce the number of zero-dose and under-immunized children globally by strengthening organizational learning capacity and facilitating high-quality evidence generation and uptake at the national, regional, and global levels to influence immunization policy and programming. The ZDLH theory of change TOC (Fig. 2) shows how global and country-level activities are aligned to achieve outcomes. CLHs also have their own theory of change (not shown here), nested within the overall TOC.

The four CLHs were established sequentially, rather than concurrently due to realities of the review and selection process:

1. Bangladesh in October 2022 [led by the International Center for Diarrhoeal Disease Research, Bangladesh (icddr,b) with Jhpiego and RedOrange Communications].
2. Mali in November 2022 [led by GaneshAID with the Center for Vaccine Development-Mali (CDV-Mali) and Faculty of Medicine and Odonto-Stomatology, University of Sciences, Techniques and Technologies of Bamako (FMOS/USTTB)].
3. Uganda in February 2023 [led by Infectious Diseases Research Collaboration (IDRC) with PATH and Makerere University School of Public Health (MakSPH)]
4. Nigeria in April 2023 [led by the African Field Epidemiology Network (AFENET) with the African Health Budget Network (AHBN)].

Findings and learnings generated through the CLHs will help identify (1) effective strategies and approaches that should continue for ZD measurement and programming, (2) how strategies and approaches are adapted to respond to determinants and barriers to reaching ZD children, and (3) what strategies and approaches are not effective and should be discontinued. Each LH consists of local partners focused on the following key objectives:

- Generate and synthesize learnings based on knowledge of the barriers to reach ZD children and apply these learnings to programme planning and tailoring equitable approaches.
- Strengthen the evidence base of effective approaches to identify and reach ZD children.
- Improve metrics, measures, and methods to access and use data on a regular basis to improve outreach to ZD children and missed communities.

The global learning partner is led by JSI Research & Training Institute, Inc. (JSI), in partnership with the International Institute of Health Management Research, New Delhi (IIHMR) and The Geneva Learning Foundation (TGLF). Their objectives include the following:

- Provide tailored and collaborative capacity strengthening, technical assistance, and mentorship as requested by CLHs, including identifying and resolving implementation challenges.
- Capture, synthesize, and disseminate the breadth of evidence and learning in a timely manner and facilitate learning and sharing within and across the learning hubs, the Alliance and other implementing partners and donors, and stakeholders within other Gavi-supported countries so that evidence is used to inform policies, guidance, and programmes.

### Theory of action

The four CLHs also have similar activities, which are designed to meet information needs within and across countries, but they are intended to be flexible and adaptable to meet the needs of the country-specific context and questions. The activity, purpose, data source, and example approaches are described in Table 2:

1. All partners conduct a **stakeholder analysis**, develop a **stakeholder engagement plan** and **country-specific learning agenda**, and **regularly engage stakeholders**. These elements ensure that all stakeholders working in the zero-dose space in the country are engaged in the implementation of learning hub activities and findings, share their own findings, help in the interpretation of evidence from the learning hubs and across partners, and define plans for evidence use.
2. The CLHs conduct at least three evidence generation activities:
  - A **rapid assessment** consisting of triangulation of existing data and primary data collection (as needed to fill gaps) to identify areas with high numbers and proportions of ZD children, barriers, existing evidence, and existing ZD data sources and gaps;
  - Activities to improve **timely monitoring** on key indicators ((Box 2); and
  - **IR of interventions** designed to reach zero-dose children using quasi-experimental designs to measure effectiveness, costs, system factors and intervention characteristics influencing adoption, implementation, and maintenance/sustainability (RE-AIM 2026).
3. **Learning products** are targeted to improve knowledge translation, communication, and use of evidence. Knowing the different stakeholders, understanding their information needs and timing of needed information, and targeting with evidence are hypothesized to be key enablers to driving evidence use (Box 3).

Box 2. **Timely monitoring**

Nigeria, the LH team developed a **decentralized immunization monitoring (DIM)** approach based on lot quality assurance sampling (LQAS), which was implemented over time to produce ward-level performance and LGA-level coverage on key immunization and behavioural data, providing information for programmatic course correction (Attahiru et al. 2025).

Box 3. **Learning products**

The Mali LH built the **Collaborative Intelligence Platform** for the National Immunization Center (CNI in French) that is a digital environment that consolidates EPI data sources, displays dashboards, hosts evidence briefs and reports, and tracks workplan implementation across partners (Gavi ZDLH 2025a).

**Table 2:** ZDLH theory of action, methods and data sources, and example approaches.

Method	Stage of results chain	Purpose	Data sources	Example approach
Stakeholder engagement	All, except impact	Co-identification of questions, information needs, and use cases Strengthen interpretation and use	Excel document mapping stakeholders with their level of influence and interest, communication approach, engagement plan Meeting notes of technical working groups and other meetings Learning Agenda documented with prioritized questions	In Bangladesh, a National Monitoring Committee, led by the Ministry of Health and Family Welfare (MOHFW), convenes regularly to inform LH recommendations and facilitate use. In Mali, a collaborative intelligence digital platform is being developed to host data, results, and activity implementation progress for all stakeholders. In Nigeria, partners are embedded in government-led working groups, and a day-long workshop using a Delphi method approach prioritized questions and identified data sources. The Uganda team regularly engages stakeholders and participates in EPI meetings.
Rapid assessment	Inputs, activities	Identify or validate the identification of geographic areas with high numbers and proportions of ZD children. Context- and intersectional- specific understanding of barriers to addressing equity and reaching ZD children Assess the ability of existing data systems to monitor and measure ZD	Literature reviews Triangulation of existing data sources Qualitative data (key informant interviews, focus groups) Data systems assessment/data quality assessment Health facility assessment	In all countries, the Behavioral and Social Drivers (BeSD) questions are included, offering a subnational view to demand-side barriers and to monitor those over time (WHO, 2022). In Bangladesh, icddr,b used lot quality assurance sampling (LQAS) to validate the identification of missed communities, following triangulation of survey and administrative data, and to identify the factors associated with being zero-dose and under-immunized (Das et al. 2024).
Monitoring key outcomes	Outputs	Improve timeliness of monitoring data and feedback loops Improved measures of DTP1 numbers immunized, coverage, and DTP1-3 dropout; gender; and demand	Routine health information systems (DHIS2) Targeted household surveys	At the global level on a semiannual basis, JSI is analyzing trends in key indicators obtained from WHO WIISEMart and triangulated with DHIS2 data from CLH partners’ target subnational area (WHO n.d.). In all countries, partners are conducting system assessments to understand how existing immunization data can monitor identification and reach of ZD children and will use this to improve monitoring. In all countries, partners are implementing targeted coverage surveys and analyzing trends in DHIS2 data. Nigeria is implementing a Decentralized Immunization Monitoring approach using LQAS at the ward level (sub-LGA) to monitor on a semiannual basis reaching ZD children and dimensions of demand and use that information at pause-and-reflect sessions to make decisions on implementation (Attahiru et al. 2025).
Implementation research	Activities, outputs, outcomes	Understand the reach, effectiveness, adoption, implementation, maintenance, and cost of interventions	Targeted household surveys Qualitative data (key informant interviews, focus groups) Health facility assessment	All partners are using quasi-experimental pre/post designs. In Bangladesh there are also comparison groups. In Mali, they are doing process evaluation. All partners are using theories of change to support decisions related to data sources and measurement and to inform interpretation of results. Three of four have integrated costing into their designs. In Mali, Uganda, and Nigeria, research will test interventions that were identified during the Gavi full portfolio funding processes in 2023.
Knowledge translation and use	Outcome, impact	To close the gap between evidence, informed decisions, and adaptive management To inform Gavi 5.0 strategy implementation monitoring To provide information to the Gavi ZD Evaluation Support adaptation and scale up of effective approaches to reach ZD	Policy briefs Reports Webinars Peer-to-peer exchanges	All partners have funding to support and implement tailored knowledge translation plans appropriate to their context. A knowledge translation toolkit resource (https://zdlh.gavi.org/resources/knowledge-translation-zero-dose-immunization-research) is available to support countries’ plans (Gavi Zero-Dose Learning Hub 2024). Bangladesh has established a hub (https://clh-immunisation-bd.org/) with findings and materials in Bangla. In Nigeria, they are holding webinar series on various topics highlighting learning hub and other partners’ results. Mali’s collaborative intelligence digital platform will house results from the learning hubs and partners. TGLF held peer-exchange events early in the project to share providers’ practices identifying and reaching ZD children in all four Country Learning Hub Countries, resulting in case studies (TGLF 2023a, 2023b).

The ZDLH global consortium focuses on four main work areas:

1. Develop and implement systems and mechanisms that **facilitate engagement, co-creation, information sharing, and collaboration** across country-level and global stakeholders. The main platform is the ZD website (https://zdlh.gavi.org), established as a subdomain of Gavi’s website, which was an important consideration to ensure sustainability. TGLF has hosted peer learning events through their digital learning platform (https://www.learning.foundation/). Other activities include webinars, meetings, and workshops.
2. **Strengthen capacity** through expert peer support tailored to partners’ specific requests and standardized tools that include guidance on key zero-dose metrics and measurement (Corrêa et al. 2024), survey tools and methods such as behavioural and social drivers (BeSD) (WHO 2022), costing methods (Levin et al. 2025), implementation research and theory-based evaluation (Stammer et al. 2025), and knowledge translation approaches (Gavi Zero-Dose Learning Hub 2024).
3. **Develop and disseminate products** such as data visualization, documents, technical briefs, and blogs that target appropriately segmented audiences to inform and influence stakeholders. An important function of the global-learning partner activities is to assess other global, regional, and country platforms with zero-dose content and ensure the cross-curation and dissemination of content. For example, many ZDLH resources have also been posted on TechNet, a global network of immunization professionals (https://www.technet-21.org/en/)
4. **Innovations,** including complexity-aware methods and peer learning, to advance knowledge on how to measure, monitor, and reduce the number of zero-dose children globally (Box 4 and 5).

Box 4. **Innovation, Outcome Harvesting**

In Uganda, the LH used a complexity-aware monitoring approach, **outcome harvesting**, to follow-up with 99 ZD children and to understand whether they were reached with immunization or not, how they were reached, and understand the barriers and enablers of vaccine uptake or not (Gavi ZDLH 2025b).

Box 5. **Innovation, Peer Learning**

To generate early learning in 2023 at the global level, The Geneva Learning Foundation (TGLF) hosted the ZDLH Inter-Country Peer Learning Exchanges (ZDLH-X). The peer learning events included three steps: (1) Registrants completed a questionnaire on local ZD challenges, practices and priorities. (2) A series of online events to share practices across IRMMA were held. (3) Follow-up knowledge translation and learning reflection events were held. Case studies of practitioners’ tailored local solutions for immunization equity were produced (TGLF 2023a, 2023b).

This paper describes a methodological framework and design of the ZDLH approach and thus did not require ethical approval as it did not involve collection of primary data from human participants.

## Results

Results of the Learning Hub initiative will mainly be measured according to the initiative’s monitoring, evaluation, and learning (MEL) plan, which has three functions: (1) monitor the implementation of the LH providers’ activities for accountability to Gavi, (2) document the measurement approaches and tools to understand how the overall learning hub initiative is working to generate and disseminate evidence and resulting in uptake of evidence at national and global levels, and (3) enable measurement to support reporting of similar data and output and outcome indicators across the country learning hubs. Country learning hubs have their own MEL plan for monitoring, learning, and accountability to Gavi. These plans are informed by and aligned with the MEL plan for the global learning partner.

Primary outcomes of the overarching global MEL plan focus on, “Timely, increased and sustainable use of evidence to improve global, regional, and country immunization programmes in alignment with Gavi 5.0 Strategy and IRMMA Framework,” which is achieved through the following outputs:

- Country learning hubs have strong networks, technical expertise, and practices.
- Cross-country evidence is generated.
- Evidence and learning available and accessible to identify and track zero-dose children and missed communities through a gender and immunization equity lens.
- Project-generated evidence and learning translated for use in local policy and programming.
- Learnings around zero-dose barriers and effective interventions communicated globally to partners, stakeholders, and immunization practitioners.

Implementation progress is monitored through regular check-ins and quarterly reports from partners. These reports are transformed on a semiannual basis into publicly-available updates on the country’s progress toward alignment with IRMMA and reporting on key monitoring indicators and progress against the project’s MEL plan (https://zdlh.gavi.org/semiannual-update) (Box 6). An Advisory Committee (AC) meets semiannually, composed of 10 global experts in immunization, monitoring, measurement, and learning. The role of the AC is to advise Gavi and specifically the MEL Team on the overall LH approach, and to provide Gavi with recommendations, guidance, and directions to consider for the ZDLH.

Box 6. **Timely learning synthesis**

The ZDLH at the global level produced five rounds of “semiannual updates” (SAR) (https://zdlh.gavi.org/semiannual-update) to synthesize learnings and evidence in a timely and accessible format. SARs draw findings from CLH quarterly reports and are refined and validated through discussions with teams. SARs consist of trends in data on three key indicators (DTP1 coverage, DTP1 number immunized, and DTP1–3 dropout); updates on intervention implementation and IR; and key insights, decisions, and use of LH results. Audience is Gavi Board and other global-level stakeholders.

The ZDLH consortia also monitor risks that may pose a threat to delivering on this learning hub agenda. Poor data quality and timely availability may limit the ability of country partners to identify and monitor reaching zero-dose children and related indicators or to share those findings at the global level. Zero-dose children as a concept is relatively new, with Pubmed showing 40 articles using this term in 2023 compared with two in 2019. Although there has been increasing alignment with the strategic approach, lack of awareness or appreciation for the zero-dose strategy or IRMMA framework may limit the implementation of zero-dose approaches. It is possible that in some contexts the zero-dose approach may not be aligned with national priorities, for example in high coverage areas, or may face competing priorities of other initiatives such as “The Big CatchUp” (O’Brien and Lemango 2023) or prioritization of new vaccine introductions such as malaria or human papilloma virus (Gavi 2016). It takes time for new investments in zero-dose interventions to intensify to the extent needed to drive change, assuming they are effective and the intensity of the interventions are sufficient. It may not be possible for the CLH methods to detect the full effect of intervention implementation given the timing of the learning hub research relative to when investments are distributed or interventions fully implemented (Gavi 2016). For example, in Mali and Uganda where learning hub partners will be conducting IR around interventions identified during the 2023 Gavi grant application process [specifically, the Full Portfolio Planning (FPP) process aligns all Gavi’s support for a country into one plan. The Equity Accelerator Fund (EAF) complements this by providing targeted resources to help countries reach zero-dose children and underserved communities through context-specific, equity-driven solutions. More information available at: https://www.gavi.org/our-support/guidelines], it takes time for funding to be disbursed to countries, then applied in support of those interventions, and more time for those activities to affect coverage outcomes (Gavi 2024b). Finally, there may be limitations with the activities in the TOC, e.g. if activities do not address actual barriers or are insufficient levers to drive change, although the CLHs are trying to address this limitation through a more adaptable model and targeted knowledge translation activities.

## Discussion

This paper describes the design and methods of a 4-year initiative (through 2025) to build the evidence base of effective approaches and costs of those approaches to identify and reach zero-dose children that are appropriate to specific contexts where zero-dose children live. This is achieved through a collaborative learning network of organizations in four countries and at the global level, with explicit mechanisms to align stakeholders and target opportunities for learning and evidence use. At the country level, learning hub partners use common methods and approaches, but these are adaptable and flexible in response to local questions and challenges. At the global level, partners engage in collaborative problem solving and learning to resolve technical challenges and synthesize and share evidence across countries. A common MEL plan will support the ability to measure the success of the Learning Hub approach to ensure that more experiences of learning health systems in are documented and shared.

It is the intention of this paper to document the design and methods of the ZDLH model, as they can inform other efforts to support nationally led efforts for learning and evidence generation of strategic priorities. Future publications and reports will address progress of implementation and achievement of the outputs and outcomes, as well as the technical results of the learning hubs’ efforts to improve monitoring and evidence generation and use. Specifically, results will demonstrate how the EPI and ministries of health can successfully (or not) design and address multiple deprivations and barriers to immunization across varied contexts and learn from this initiative about how to better monitor and learn about strategy translation at subnational levels.

## Data Availability

No new data were generated or analysed in support of this research.

## Reflexivity statement

Two of the seven coauthors identify as men. Coauthors represent three different countries of origin, one from an upper-middle-income country and the others from upper income countries. We recognize that our professional roles, institutional affiliations, and prior experiences have shaped the design, interpretation, and presentation of this work. The Zero-Dose Learning Hub (ZDLH) initiative described in this paper was designed with broad input from diverse partners in many countries with variation in the World Bank income groups.
